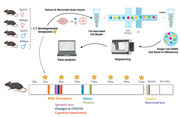# Developing a transcriptomic atlas of Alzheimer's Disease progression in the Tg2576 mouse model using single‐cell RNA‐sequencing technology

**DOI:** 10.1002/alz70855_097892

**Published:** 2025-12-23

**Authors:** Kelly Marie Johns, Giorgia Caspani, Wing Yan Leung, Chaahat S.B. Singh, Maria‐Elizabeth Baeva, Cheryl Pfeifer, Wilfred A Jefferies

**Affiliations:** ^1^ University of British Columbia, Vancouver, BC, Canada

## Abstract

**Background:**

Alzheimer's disease (AD) is a progressive neurodegenerative disorder marked by cognitive decline, ultimately leading to dementia and death. While the amyloid hypothesis has historically guided AD research, emerging evidence suggests alternative mechanisms, including vascular dysfunction. Angiogenesis, the formation of new blood vessels, is increasingly implicated in early AD pathology, yet transcriptomic insights remain limited. To address this gap, we generated the first single‐cell transcriptomic profile of AD progression in a mouse model, aiming to identify novel molecular mechanisms and potential therapeutic targets.

**Method:**

We analyzed brain tissue from Tg2576 AD model and control mice (*N* = 28, both sexes) at six developmental timepoints. Single‐cell RNA sequencing was performed using 10X Genomics technology, followed by pathway analysis to identify transcriptional changes associated with AD pathology.

**Result:**

Pathway enrichment analysis in 6‐ and 9‐month‐old AD mice revealed significant upregulation of angiogenesis (*p* = 3.37E‐02), vasculature development (*p* = 3.71E‐02), and amyloid‐beta formation (*p* = 1.49E‐02).

**Conclusion:**

These findings provide a single‐cell resolution view of AD‐related transcriptional changes, supporting a ‘vascular angiogenesis model’ of AD. Disruptions in blood‐brain barrier integrity due to aberrant neoangiogenesis may drive amyloid‐beta accumulation, contributing to disease progression. This study underscores the therapeutic potential of targeting vascular dysfunction in AD.